# Cell cycle protein Bora serves as a novel poor prognostic factor in multiple adenocarcinomas

**DOI:** 10.18632/oncotarget.16631

**Published:** 2017-03-28

**Authors:** Qiong-Xia Zhang, Rui Gao, Jin Xiang, Zhong-Yu Yuan, Yuan-Min Qian, Min Yan, Zi-Feng Wang, Quentin Liu, Hai-Dong Zhao, Chang-Hong Liu

**Affiliations:** ^1^ Sun Yat-Sen University Cancer Center, The Second Affiliated Hospital, Dalian Medical University, Dalian 116044, China; ^2^ Department of Oncology, The First Affiliated Hospital of Guangdong Pharmaceutical University, Guangzhou 510060, China; ^3^ Department of Pathology, Sun Yat-Sen University Cancer Center, Guangzhou 510060, China; ^4^ Department of Gynecology, Guangzhou Women and Children's Medical Center, Guangzhou Medical University, Guangzhou 510060, China

**Keywords:** Bora, prognosis, cancer, biomarker

## Abstract

Cell cycle protein Bora has been identified to integrate the functions of three major mitotic kinases: Cyclin-dependent kinase-1, Polo-like kinase-1, and Aurora A kinase. Overexpression of Bora disrupts spindle assembly and causes genomic instability. However, the clinical relevance of Bora in cancer remains unclear. In this study, we examined the expression of Bora and its association with clinical characteristics in breast (*n* = 538), lung (*n* = 144) and gastric (*n* = 77) adenocarcinomas. We found that Bora was overexpressed in primary breast cancer tissues compared to paired non-cancerous tissues. Bora overexpression was observed at a higher proportion in triple-negative breast cancer (TNBC, 77.63%) compared with non-TNBC subtypes (42.76%, *P* < 0.0001). Kaplan-Meier survival analysis indicated that Bora overexpression was associated with unfavourable overall survival (OS, *P* < 0.0001) and disease-free survival (DFS, *P* = 0.007) in breast cancer. In addition, Bora subclassified patients with distinct clinical outcomes in both stages (II/III) and subtypes (HR+, HER2+) of breast cancer. Consistently, Bora was associated with adverse prognosis in lung (*P* = 0.005 for OS and DFS *P* = 0.001 for DFS) and gastric adenocarcinomas (*P* < 0.0001 for OS, and *P* < 0.0001 for DFS). Moreover, Bora was positively correlated with proliferation index Ki67 in breast and gastric cancer (*P* < 0.001, *P* = 0.005, respectively). Multivariate analyses further revealed that Bora was an independent prognostic parameter for OS and DFS in all three types of adenocarcinomas. In conclusion, our findings demonstrated that Bora was overexpressed and served as an independent biomarker for poor prognosis in multiple adenocarcinomas.

## INTRODUCTION

Cell cycle deregulation, a fundamental hallmark of cancer, has been recognized as a driving force for cancer proliferation [1, 2]. Cell cycle regulators have been intensively studied as both biomarkers and therapeutic targets. Core regulators, such as p53, p21, p27, pRb, separase cyclin D, cyclin E, Aurora A and Polo-like kinase-1 (Plk1) are aberrantly expressed in various types of cancers, associating with tumor progression and prognosis [3–10]. Above them, Aurora A and Plk1 are key regulators of cell-cycle processes, including mitotic entry, centrosome maturation, spindle assembly and sister chromatid cohesion. Aberrant activation of either Aurora A or Plk1 can promote the development of cancer and they represent promising targets for anticancer therapeutics [10–12].

*Bora* (also known as C13orf134 and FLJ 22624) is located at Chr13q22.1, a malignant susceptibility locus in cancer [13]. Bora is originally identified as a cell cycle protein interacting with Aurora A in *Drosophila* [14]. Bora expression is low in G1/S boundary but increases in late S phase, peaks in the G2 phase and is degraded during mitosis [15–17]. Specifically, Bora interacts with Plk1 and controls the accessibility of its activation loop for phosphorylation and activation by Aurora A, thus promote mitotic entry [15, 18, 19]. Low levels of Bora is also required to sustain Plk1 activity during mitosis [20]. Recent studies highlight the role of Bora in G2-M transition, which is critical for the DNA damage dependent checkpoint that guards genomic stability [18, 21–23]. Therefore, the Bora-Aurora A-Plk1 axis is critical for coordinating cell cycle progression and genomic stability, two key processes that are involved in cancer initiation and progression. However, the expression and the clinicopathological significance of Bora in cancer remains unclear.

In the present study, we examined Bora expression and its clinicopathological significances in breast, lung and gastric adenocarcinomas. We found that cell cycle protein Bora was highly expressed in primary breast cancer tissues compared to paired non-cancerous tissues. Further analysis indicated that high expression of Bora was associated with poor overall survival and disease-free survival in breast, lung and gastric adenocarcinomas. Moreover, Bora overexpression was associated with poor prognosis in distinct clinical stages (II/III) and subtypes (HR+, HER2+) of breast cancer. Multivariate analysis further demonstrated that high expression of Bora was an independent prognostic parameter for both OS and DFS in all the three types of adenocarcinomas.

## RESULTS

### Bora was overexpressed in breast cancer tissues and enhanced cell proliferation

**Figure 1:**
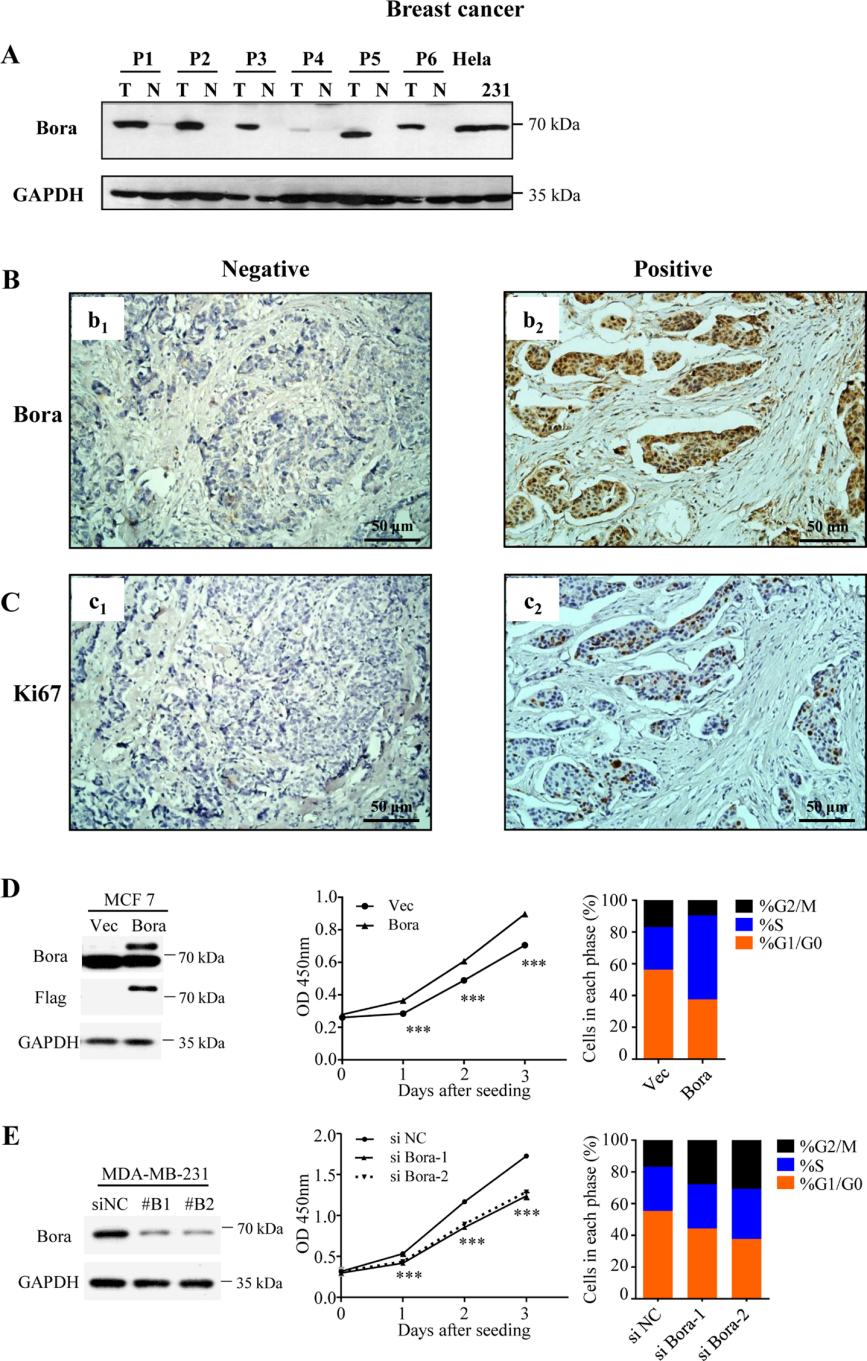
Bora was overexpressed in breast cancer tissues and enhanced cell proliferation (**A**) Western blotting analysis of Bora expression in representative primary breast cancer tissues (T) and normal breast tissues (N). GAPDH was used as a loading control. Lysates of Hela and MDA-MB-231 cells were served as positive controls. (B and C) Representative immunohistochemical (IHC) staining for Bora (**B**) and Ki67 (**C**) in breast cancer tissues. Scale bars, 50 μm. **(D**) Empty vector (Vec) or Flag-Bora (Bora) overexpressed MCF-7 cells were collected for western blotting analysis (Left). Cell proliferation was assessed every day for 3 days using CCK-8 assays. Results were shown as the mean ± S.D. from three independent experiments. ***P < 0.0001, two-sided *t* test (Middle). And the cell-cycle parameters were analyzed by flow cytometry (Propidium iodide). Data were shown as the mean value of three independent experiments (Right). (**E**) MDA-MB-231 cells were transfected with control (siNC) or two siRNAs against Bora (siBora-1 and -2). Depletion efficiency was validated by Western blot (Left). Cell proliferation after siRNA transfection was assessed every day for 3 days using CCK-8 assays. Results were shown as the mean ± S.D. from three independent experiments. ***P < 0.0001, one-way ANOVA (Middle). And the cell cycle was analyzed at 48 h after transfection. Data were shown as the mean value of three independent experiments (Right).

To determine whether Bora was aberrantly expressed in cancer, we compared Bora expression in paired breast cancer and non-cancerous tissues. In all the six paired samples, Bora was highly expressed in breast cancer tissues compared with paired non-cancerous tissues (Figure 1A). Immunohistochemistry (IHC) staining of Bora in breast cancer tissues indicated that Bora was overexpressed in the cytoplasm of the breast cancer cells (Figure 1B, b_2_). Furthermore, High expression of Bora was positively correlated with Ki67 the index of cell proliferation (Figure 1C). In conclusion, Bora was overexpressed in breast cancer and associated with cancer proliferation.

We then examined the functional association of Bora and cell proliferation. Consistently, we found that overexpression of Bora (Figure 1D, Left) in MCF-7 cells increased p-Plk1 (Supplementary Figure 1A) and enhanced cell proliferation (Figure 1D, Middle). Whereas knockdown of Bora (Figure 1E, Left) in MDA-MB-231 cells significantly reduced cell proliferation (Figure 1E, Middle). Flow cytometric analysis indicated that Bora overexpression in MCF-7 cells resulted in a significant decrease of cells in G0/G1 phase, and a substantial increase of cells in S phase (Figure 1D, Right). In contrast, knockdown of Bora in MDA-MB-231 cells resulted in cell cycle arrested in G2/M phase (Figure 1E, Right). Interestingly, TUNEL assay indicated that neither overexpression nor knockdown of Bora caused DNA damage (Supplementary Figure 1B). Thus, Bora was functionally involved in cell cycle process and enhanced proliferation in breast cancer cells.

### Systematic analyses of Bora expression and clinical features

To examine the clinical relevance of Bora in breast cancer, the clinical features, including age, clinical stage, tumor classification, node classification, P53 and Ki67 were analysed with Bora expression in breast cancer (Table 1). Breast cancer patients were classified into high (283/538, 52.60%) and low (255/538, 47.40%) expression subgroups according to the cut-off score by Receiver Operating Characteristic (ROC) curve analysis. Bora expression was positively correlated with Ki67 level (*P* < 0.001) and molecular subtypes (*P* < 0.001), but not other clinical characteristics. Detailed analysis was performed in breast cancer subtypes defined by IHC: HR+ (ER+ and/or PR+ and Her2-), HER2+ and TNBC (ER- and PR- and HER2-) [24]. High expression of Bora was found at a higher proportion in TNBC (77.63%) compared with non-TNBC (42.76%, *P* < 0.001) subtypes.

**Table 1:** Bora expression and patients’ characteristics

	Breast cancer				Lung cancer				Gastric cancer			
Variable	*N*	low	high	*P*^a^	*N*	low	high	*P*^a^	*N*	low	high	*P*^a^
All patients	538	283	255		144	65	79		77	25	52	
**Age (years**)												
≤ 48^b^ (59^c^,55^d^)	276	142	134	0.58	67	30	37	0.94	41	16	25	0.19
> 48 (59,55)	262	141	121		77	35	42		36	9	27	
**Gender**												
Male	0				104	47	57	0.98	44	13	31	0.53
Female	538	283	255		40	18	22		33	12	21	
**Clinical stage**												
I	107	65	42		47	24	23		7	5	2	
II	259	135	124	0.12	30	15	15	0.56	8	4	4	0.05
III	168	83	85		64	25	39		42	12	30	
IV	2	0	2		3	1	2		20	4	16	
Missing cases	2											
T **classification**												
T_1_	196	111	85		13	4	9		3	2	1	
T_2_	258	134	124	0.37	100	46	54	0.70	9	5	4	0.17
T_3_	44	19	25		26	13	13		42	13	29	
T_4_	39	19	20		5	2	3		23	5	18	
Missing cases	1											
**N classification**												
N_0_	236	126	110		63	33	30		10	6	4	
N_1_	158	91	67	0.19	23	11	12	0.33	35	13	22	0.09
N_2_	86	37	49		56	20	36		21	4	17	
N_3_	56	29	27		2	1	1		11	2	9	
Missing cases	2											
P53												
Negative	361	193	168	0.36	93	50	43	0.005	32	12	20	0.43
Positive	157	77	80		51	15	36		45	13	32	
Missing cases	20											
**Ki67**												
Negative	178	141	37	< 0.001	72	37	35	0.13	29	15	14	0.005
Positive	350	135	215		72	28	44		48	10	38	
Missing cases	10											
**Intrinsic type**												
HR(+)	315	182	133									
HER2(+)	144	82	62	< 0.001								
TNBC	76	17	59									
Missing cases	3											

^a^Chi-square test, ^b^median age in breast cancer, ^c^median age in lung cancer, ^d^median age in gastric cancer.

### High Bora expression indicated poor survival outcome in breast cancer

**Figure 2:**
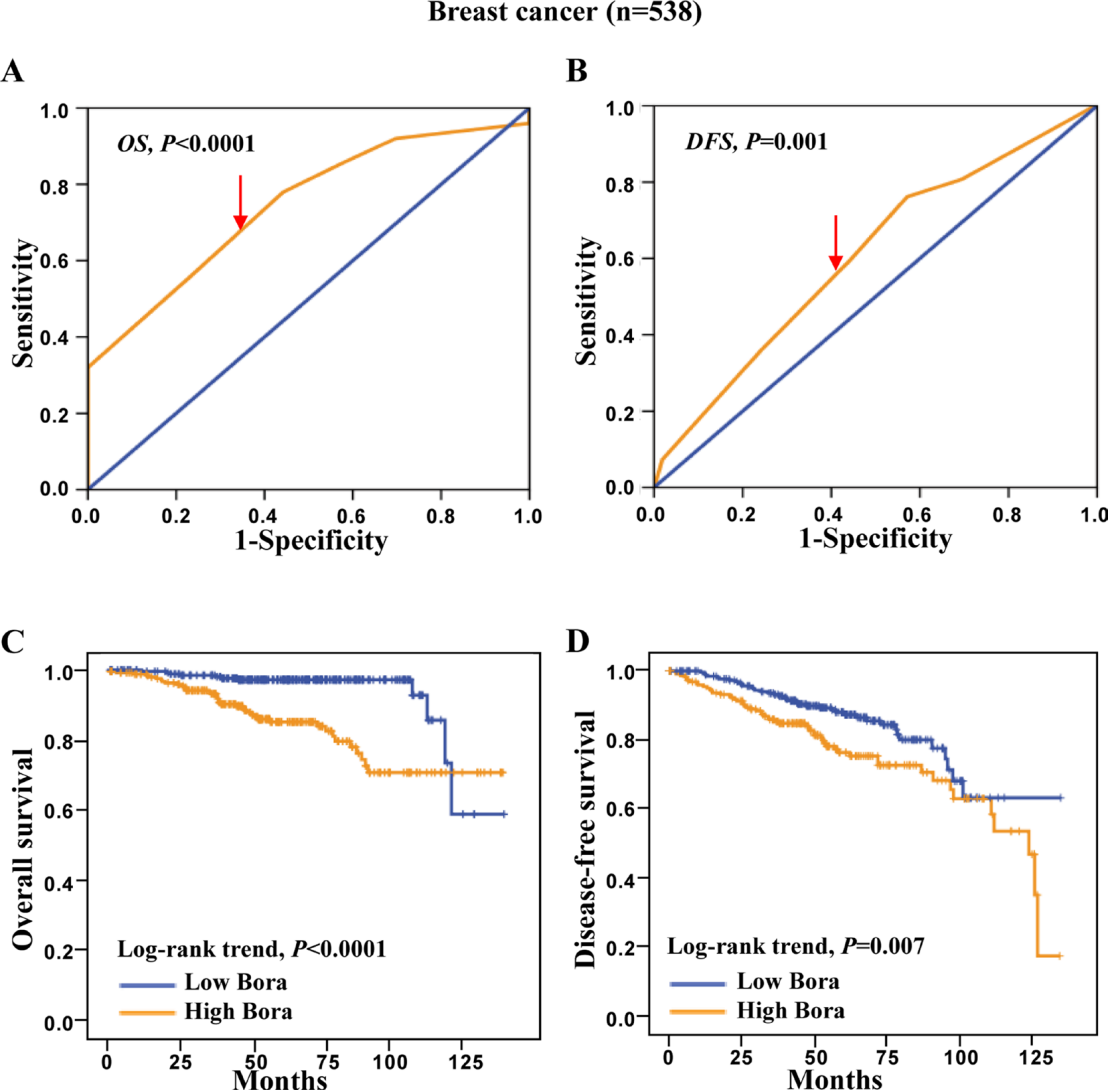
ROC curves and Kaplan-Meier survival analysis in breast cancer (**A** and **B**) Bora cut-off point for overall survival (A) and disease-free survival (B) in breast cancer. Bora cut-off score for overall survival and disease-free survival was 4.83 and 4.72, respectively. (**C** and **D**) Kaplan-Meier curves for overall survival (C) and disease-free survival (D) of breast cancer patients according to Bora expression status. The *P*-values were determined using the log-rank test.

To assess the clinical significance of Bora in breast cancer, survival analysis was carried out in the cohort of breast cancer (*n* = 538). ROC curve analysis was employed to determine cut-off point for Bora expression in overall survival (OS) and disease free survival (DFS) analysis (Figure 2A and 2B, respectively). We set Bora expression score 5 (≥ 5 VS. < 5) as the cut-off for OS (4.83, *P* < 0.0001) and DFS (4.72, *P* = 0.001). Patients with high Bora expression showed a significantly worse prognosis for both OS and DFS than those with low expression (*P* < 0.0001, Figure 2C; *P* = 0.007, Figure 2D). These results indicated that Bora predicted poor prognosis in breast cancer.

### Bora significance in clinical stages and subtypes of breast cancer

**Figure 3:**
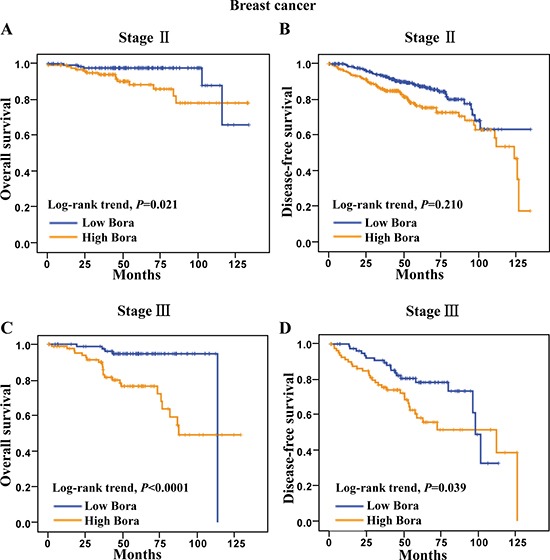
Survival analysis of Bora in clinical stage II and III breast cancer patients (**A** and **B**) Kaplan-Meier curves for overall survival (A) and disease-free survival (B) of breast cancer patients in clinical stage II. (**C** and **D**) Kaplan-Meier curves for overall survival (C) and disease-free survival (D) of breast cancer patients in clinical stage III. The *P*-values were determined by the log-rank test.

To further analyze the significance of Bora in breast cancer subsets, we performed survival analysis in different clinical stages. We excluded the stage I and IV subsets due to the high survival rate (107/107, 100%) of stage I and limited case number (*N* = 2) of stage IV patients. The results showed that high Bora expression was significantly associated with poor OS (*P* = 0.021, Figure 3A), but not DFS (*P* = 0.210, Figure 3B) in stage II patients. In contrast, high expression of Bora indicated significantly poor outcomes in both OS (*P* < 0.0001, Figure 3C) and DFS (*P* = 0.039, Figure 3D) of stage III patients.

**Figure 4:**
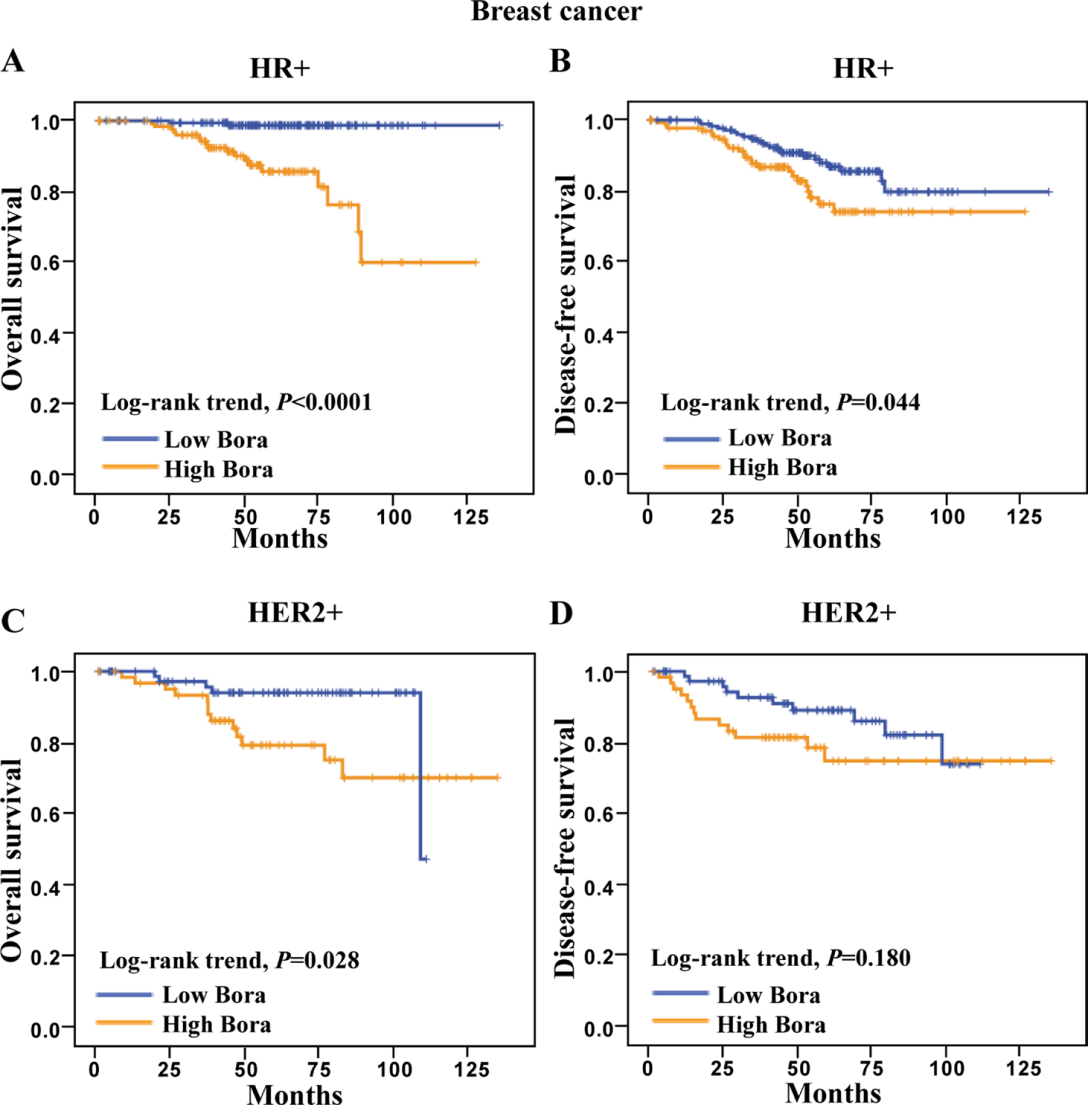
Survival analysis of Bora in HR+ and HER2+ breast cancer patients (**A** and **B**) Kaplan-Meier curves for overall survival (A) and disease-free survival (B) of HR+ breast cancer patients. (**C** and **D**) Kaplan-Meier curves for overall survival (C) and disease-free survival (D) of HER2+ breast cancer patients. The *P*-values were determined by the log-rank test.

We next determined the clinical significance of Bora in distinct molecular subtypes (HR+, HER2+ and TNBC) of breast cancer [24]. We found that high Bora expression was associated with poor clinical outcomes in both HR+ and HER2+ subtypes, but not TNBC. Specifically, high Bora expression indicated significantly poor OS (*P* <0.0001, Figure 4A) and DFS (*P*=0.044, Figure 4B) in HR+ patients. In HER2+ breast cancer patients, high expression of Bora was associated with poor OS (*P*=0.028, Figure 4C) but not DFS (*P* = 0.180, Figure 4D). However, Bora did not serve as a prognostic factor in TNBC (data not shown), and larger case numbers might be needed to address this issue. These results indicated that Bora predicted poor prognosis in distinct breast cancer subtypes.

### High bora expression indicated poor survival outcome in lung adenocarcinoma

**Figure 5:**
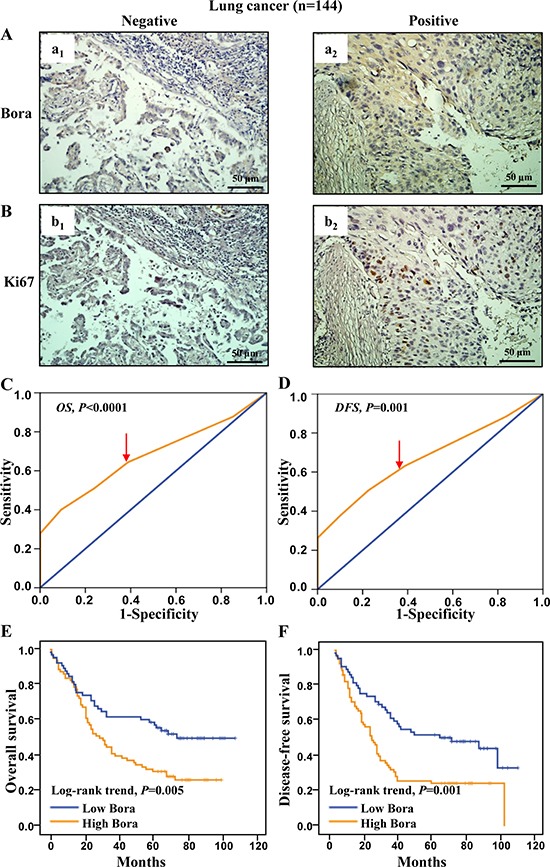
Bora expression and survival analysis in lung cancer (**A** and **B**) Representative IHC staining for Bora (A) and Ki67 (B) in lung cancer tissues. Scale bars, 50 μm. (**C** and **D**) Bora cut-off point for overall survival (C) and disease-free survival (D) in lung cancer. Bora cut-off score for overall survival and disease-free survival were 2.75 and 2.70, respectively. (**E** and **F**) Kaplan-Meier curves for overall survival (E) and disease-free survival (F) of lung cancer patients according to Bora expression. The *P*-values were determined by the log-rank test.

To study the clinical significance of Bora in lung cancer, we examined Bora expression in lung adenocarcinoma. The expression of Bora (Figure 5A) and Ki67 (Figure 5B) was positively correlated. Patients were defined as high (79/144, 54.86%) and low Bora (65/144, 45.14%) expression subgroups based on the ROC-derived Bora cut-off score 3 for both OS (2.75, *P* < 0.0001, Figure 5C) and DFS (2.70, *P* = 0.001, Figure 5D). Correlation analysis demonstrated that Bora expression was positively correlated with P53 level (*P* = 0.005, Table 1). The percentage of high Bora expression in Ki67 positive lung cancer group (44/72, 61.11%) was higher than that of Ki67 negative group (35/72, 48.61%). Furthermore, patients with high Bora expression exhibited significantly worse OS (*P* = 0.005, Figure 5E) and DFS (*P* = 0.001, Figure 5F) than those with low Bora expression. These results indicated that Bora was also an adverse prognostic factor for lung adenocarcinoma.

### Bora expression and survival analysis in gastric cancer

**Figure 6:**
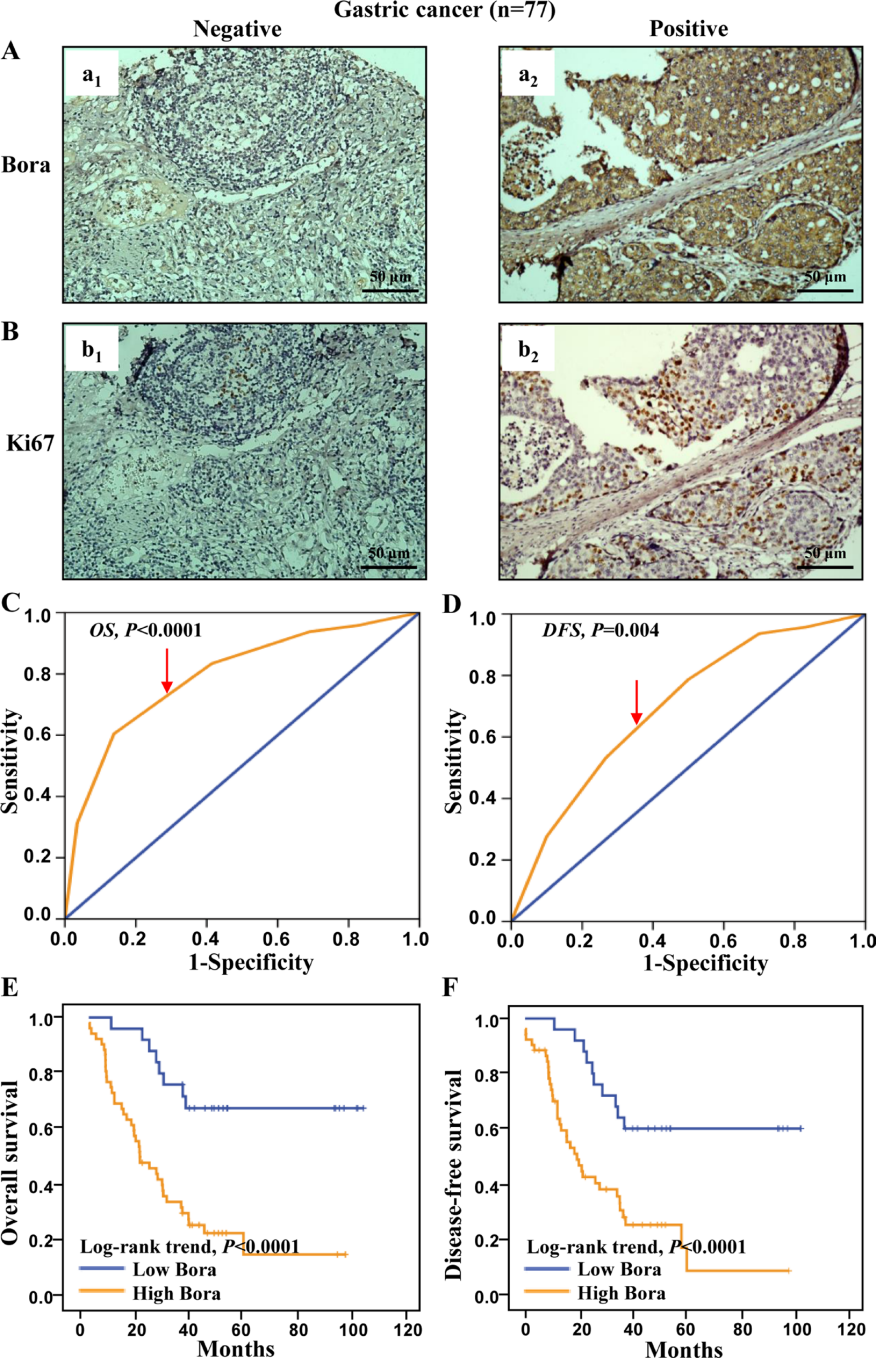
Bora expression and survival analysis in gastric cancer (**A** and **B**) Representative IHC staining for Bora (A) and Ki67 (B) in gastric cancer tissues. Scale bars, 50 μm. (**C** and **D**) Bora cut-off point for overall survival (C) and disease-free survival (D) in gastric cancer. Bora cut-off score for overall survival and disease-free survival were 4.85 and 4.95, respectively. (**E** and **F**) Kaplan-Meier curves for overall survival (E) and disease-free survival (F) of gastric cancer patients according to Bora expression. The *P*-values were determined by the log-rank test.

We further assessed the clinical significance of Bora in gastric adenocarcinoma. Consistent with the results in breast cancer, the expression of Bora (Figure 6A) and Ki67 (Figure 6B) was positively correlated. Patients were grouped into high (cut-off score 5, 52/77, 67.53%) and low (25/77, 32.47%) Bora expression subsets according to the ROC-based analyses for OS (4.83, *P* < 0.0001, Figure 6C) and DFS (4.72, *P* = 0.004, Figure 6D). Correlation analysis demonstrated that Bora expression was positively correlated with Ki67 level (*P* = 0.005, Table 1) as well as advanced clinical stage (marginal *P*=0.05, Table 1). Patients with high expression of Bora were associated with poor OS (*P* < 0.0001, Figure 6E) and DFS (*P* < 0.0001, Figure 6F) compared to those with low Bora expression, indicating that Bora was an adverse prognostic factor for gastric cancer.

### Multivariate analysis and clinical outcome in three types of adenocarcinomas

To avoid the bias caused by univariate analysis, multivariate Cox analysis was performed in in three types of adenocarcinomas (Tables 2–4). Specifically, significant clinical factors for OS were tumor size and clinical stage (*P* = 0.011 and 0.009, respectively), whereas tumor size (*P* = 0.003), clinical stage (*P* = 0.009), PR (*P* = 0.001) and Ki67 (*P* = 0.042) were significant factors for DFS in breast cancer. Bora was indeed an independent factor to predict the poor prognosis for both OS (HR: 4.317, CI: 2.147 to 8.680, *P* < 0.001) and DFS (HR: 1.686, CI: 1.131 to 2.511, *P* = 0.010) in breast cancer (Table 2). Both clinical stage (*P* < 0.001) and Ki67 (*P* = 0.001 for OS, *P* = 0.020 for DFS) were linked to OS and DFS in lung adenocarcinoma. Consistently, Bora was also an independent biomarker for adverse prognosis of OS (HR: 1.838, CI: 1.919 to 2.836, *P* = 0.006) and DFS (HR: 1.671, CI: 1.088 to 2.565, *P* = 0.019) in lung adenocarcinoma (Table 3). In addition, Table 4 showed that Bora was an independent biomarker for poor prognosis of OS (HR: 4.360, CI: 2.019 to 9.414, *P* < 0.001) and DFS (HR: 3.552, CI: 1.698 to 7.431, *P* = 0.001) in gastric adenocarcinoma. In conclusion, Bora was an independent biomarker for poor prognosis in breast, lung, and gastric adenocarcinomas.

**Table 2:** Multivariate cox proportional hazard analysis of prognostic variables in breast cancer patients

	For Overall Survival			For Disease-Free Survival		
Variable	HR	95% CI	*P*^a^	HR	95% CI	*P*^a^
**Age** > 48^b^ (vs. ≤ 48 y)	0.972	0.542 to 1.744	0.924	0.600	0.397 to 0.906	0.015
**Tumor size** > 2 (vs. ≤ 2 cm)	1.986	1.174 to 3.359	0.011	1.772	1.216 to 2.581	0.003
**Clinical stage** IV + III (vs. II+ I)	2.304	1.236 to 4.298	0.009	1.777	1.156 to 2.732	0.009
**ER** Positive (vs. Negative)	0.568	0.151 to 2.138	0.403	0.749	0.336 to 1.666	0.478
**PR** Positive (vs. Negative)	1.524	0.436 to 5.325	0.509	0.476	0.308 to 0.736	0.001
**HER2** Positive (vs. Negative)	1.807	0.936 to 3.486	0.078	1.109	0.671 to 1.834	0.686
**P53** Positive (vs. Negative)	0.917	0.478 to 1.758	0.793	0.909	0.577 to 1.430	0.679
**Ki67** Positive (vs. Negative)	1.708	0.817 to 3.568	0.155	1.614	1.018 to 2.559	0.042
**Bora** High expression(vs. Low expression)	4.317	2.147 to 8.680	< 0.001	1.686	1.131 to 2.511	0.010

^a^log-rank test, ^b^median age; ER, estrogen receptor; PR, progesterone receptor; HER2, human epidermal growth factor receptor 2;

HR, hazard ratio; CI, confidence interval.

**Table 3:** Multivariate cox proportional hazard analysis of prognostic variables in lung cancer patients

	For Overall Survival			For Disease-Free Survival		
Variable	HR	95% CI	*P*^a^	HR	95% CI	*P*^a^
**Age** > 59^b^ (vs. ≤ 59 y)	1.283	0.834 to 1.975	0.257	1.120	0.738 to 1.699	0.594
**Gender** Female (vs. Male)	0.657	0.400 to 1.077	0.096	0.792	0.494 to 1.272	0.336
**Clinical stag**e IV + III (vs. II + I)	2.813	1.811 to 4.369	< 0.001	2.564	1.672 to 3.932	< 0.001
**P53** Positive (vs. Negative)	1.011	0.643 to 1.590	0.961	1.288	0.830 to 1.998	0.259
**Ki67** Positive (vs. Negative)	2.157	1.379 to 3.374	0.001	1.661	1.085 to 2.542	0.020
**Bora H**igh expression(vs. Low expression)	1.838	1.919 to 2.836	0.006	1.671	1.088 to 2.565	0.019

^a^log-rank test, ^b^median age; HR, hazard ratio; CI, confidence interval.

**Table 4:** Multivariate cox proportional hazard analysis of prognostic variables in gastric cancer patients

	For Overall Survival			For Disease-Free Survival		
Variable	HR	95% CI	*P*^a^	HR	95% CI	*P*^a^
**Age** > 55 ^b^ (vs. ≤ 55 y)	1.564	1.823 to 2.971	0.172	1.343	0.695 to 2.597	0.381
**Gende**r Female (vs. Male)	1.627	0.888 to 2.981	0.115	1.724	0.926 to 3.210	0.086
**Clinical stage** IV + III (vs. II+ I)	2.950	1.242 to 7.005	0.014	1.319	0.580 to 3.003	0.509
**P53** Positive (vs. Negative)	0.890	0.472 to 1.678	0.718	1.189	0.624 to 2.266	0.599
**Ki67** Positive (vs. Negative)	1.427	0.670 to 3.040	0.356	1.054	0.520 to 2.133	0.885
**Bor**a High expression(vs. Low expression)	4.360	2.019 to 9.414	< 0.001	3.552	1.698 to 7.431	0.001

^a^log-rank test, ^b^median age; HR, hazard ratio; CI, confidence interval.

## DISCUSSION

We have made novel findings in the present study (1) Bora was highly expressed in breast cancer tissues, compared to paired non-cancerous tissues. (2) Patients with high Bora expression displayed poor OS and DFS in breast, lung and gastric adenocarcinomas. (3) High expression of Bora was positively correlated with the cell proliferation index Ki67. (4) Bora was associated with poor prognosis in breast cancer patients of distinct clinical stages II/III and subtypes (HR+, HER2+). (5) Bora expression was an independent prognostic parameter for OS and DFS in all three types of adenocarcinomas. Taken together, our findings provide evidence that high Bora expression indicate poor prognosis in breast, lung and gastric adenocarcinomas.

As a key cell cycle protein, Bora facilitates the recovery of G2/M phase checkpoint through Aurora A mediated phosphorylation and activation of Plk1 kinase [15, 18]. The G2/M checkpoint prevents cells from entering mitosis upon DNA damage, providing an opportunity for DNA damage repairing [25]. Our results showed that Bora was highly expressed in primary breast cancer tissues compared to non-cancerous tissues. Particularly, a higher proportion of Bora expression was observed in TNBC subtype, which displayed significantly higher rates of local and distant recurrence [26]. In addition, high expression of Bora was positively correlated with the cell proliferation index Ki67, enhancing cell proliferation and cell cycle progression. Our data demonstrated that the cell cycle protein Bora was overexpressed in cancer tissues and associated with adverse clinical outcomes.

Currently, little is known about the potential prognostic biomarkers across multiple adenocarcinomas. Here, Bora serves as a potential biomarker for poor prognosis across three types of adenocarcinomas. High expression of Bora was associated with poor outcome independent of known prognostic markers in breast, lung and gastric adenocarcinomas. In addition, high expression of Bora predicted poor OS and DFS in breast cancer patients with HR+ or HER2+, indicating that Bora could be a novel prognosis factor in addition to ER, PR and HER2 to accurately stratify patients. These findings hold significant clinical application in predicting prognosis and identifying high risk patients, and promote future studies in exploring Bora as a therapeutic target in cancer.

Recently, a large number of inhibitors for Aurora A and Plk1 kinases are being evaluated as anticancer drugs in Phase I or Phase II clinical trials, such as Aurora A kinase inhibitors: MLN8054 (Phase I), MLN8237 (Phase II), ENMD-2076 (Phase II), MP529 (preclinical) and Plk1 inhibitors: BI2536 (Phase II), BI6727 (Phase II), GSK-461364 (Phase I) [27]. However, both kinase inhibitors only showed modest bioactivity against solid tumors [28, 29]. Thus, it's urgently needed to identify novel candidate biomarkers and targets that can enhance the efficacy of existing Aurora A or Plk1 inhibitors. Bora, which links these two kinases to promote cell cycle progression [18], might serve as a potential candidate. Indeed, Bora deletion greatly reduces phosphorylation of Plk1 by Aurora A, reduces the inter-kinetochore tension and activates the tension-sensitive spindle checkpoint [14, 15], leading to cell cycle arrest or mitotic delay [16, 17]. Our study showed that Bora was overexpressed in multiple adenocarcinomas, knockdown of Bora impaired cell proliferation and arrested cell cycle in G2/M phase. Further studies are required to evaluate the anti-tumor effects of Aurora A and Plk1 inhibitors after Bora blockade.

In conclusion, to our knowledge, this is the first study reporting that Bora was an independent biomarker for adverse overall survival and disease-free survival in breast, lung and gastric adenocarcinomas. Bora, a critical player of the Aurora A-Bora-Plk1 axis in controlling cell cycle progression, is a candidate prognostic biomarker and potential therapeutic targets in cancer.

## MATERIALS AND METHODS

### Patients and eligibility

Formalin-fixed and paraffin-embedded tissues from 538 breast cancer patients, 144 lung cancer patients and 77 gastric cancer patients, who underwent initial surgical resection between 1999 and 2007, were randomly selected from the Department of Pathology, Cancer Center and the Second Affiliated Hospital of Sun Yat-sen University (Guangzhou, China). Patients satisfying the following criteria were selected to participate: (1) over 5-year follow up period; (2) microscopically confirmed adenocarcinoma; (3) no prior radiation therapy history; (4) receiving unified regimen as first-line chemotherapy after resection of primary tumors. All tumors were classified and staged according to the revised guidelines advocated by the International Union against Cancer. Demographic and detailed clinical characteristics of all patients were presented in Table 1. The follow-up deadline was summarized in January 2012 (OS was defined as the time from diagnosis to the date of death or when censused at the latest date if patients were still alive; DFS was defined as the time from diagnosis to the date of local failure/distant metastasis or the date of death or when censused at the latest date). The study obtained informed consent from all patients at their recruitment time and approval from the Institute Research Ethics Committee of Sun Yat-sen University.

### Tissue microarrays

Construction of tissue microarrays (TMAs) were performed as previously described [30]. Briefly, the formalin-fixed, paraffin-embedded tissue blocks and the corresponding histological H&E stained slides were overlaid for tissue TMA sampling. In view of tumor heterogeneity, triplicate 0.6mm-diameter cylinders of tissue were punched from selected tumor areas of individual donor tissue block and re-embedded into a recipient paraffin block at defined position, using a tissue arraying instrument (Beecher Instruments, Silver Spring, MD). The TMA block contained 538 breast cancer samples, 144 lung cancer samples and 77 gastric cancer samples. Subsequently, multiple sections (5μm thick) were cut from the TMA block and mounted on microscope slides. H&E staining was used to one section from the tissue array to confirm that the punches contained tumor region.

### Immunohistochemistry

The IHC studies of ER, PR, HER2, Ki67, P53 and Bora were performed using a standard of two-step technique. The TMA slides were dried at 65°C for 4h, dewaxed in xylene, rehydrated through graded alcohol, and immersed in 3% hydrogen peroxide for 20 min to block endogenous peroxidase activity. An antigen retrieval process was accomplished in a microwave oven with 10 M citrate buffer (pH 6.0), for 15 min. The slides were incubated with 3% bovine serum albumin at room temperature (RT) for 30 min to reduce nonspecific reaction. Subsequently, the TMA slides were incubated with the antibody against ER (monoclonal rabbit; 1:100; Thermo, SP1), PR (monoclonal mouse; 1:100; Dako, PgR 636), HER2 (monoclonal rabbit; 1:100; ZA-0023), Ki67 (monoclonal mouse; 1:150; Dako, MIB-1), P53 (monoclonal mouse; 1:500; Dako, clone D07) and Bora (polyclonal rabbit; 1:100; sigma, SAB3500025), overnight at 4°C, respectively. After three times of rinsing with 0.01M phosphate-buffered saline (PBS, pH = 7.4) for 10 minutes, the detection of the primary antibody was achieved with a secondary antibody (Envision, Dako, Denmark) for 30 minutes at RT, and stained with DAB (3,3-diaminobenzidine) after washed in PBS again. Finally, the sections were counterstained with Mayer's hematoxylin, dehydrated, and mounted. A negative control was obtained by replacing the primary antibody with a normal murine IgG.

### Immunohistochemical analysis evaluation

The brown granules in cytoplasm were considered as Bora positive staining. We scored the staining intensity as follows: 0, no staining; 1+, mild staining; 2+, moderate staining; 3+, intense staining. The area of staining was evaluated as follows: 0, no staining of cells in any microscopic fields; 1+, < 25% of tissue stained positive; 2+, 25–50% stained positive; 3+, 50–75% stained positive; 4+, > 75% stained positive. Bora expression was evaluated by combined assessing of staining intensity and extension. The minimum score when summed (intensity + extension) was 0, and the maximum was 7. Two independent pathologists scored each section, and agreement on staining intensity was 89.6% (482/538) in breast cancer, 86.8% (105/144) in lung cancer and 90.9% (70/77) in gastric cancer. The sections that was not concordance were reassessed by both of them.

ER and PR positivity was defined as ≥ 10% positive tumor cells with nuclear staining [31]. HER2+ positivity was defined as IHC scored of 3+ [32]. Cells stained for Ki67 and P53 were counted and expressed as a percentage. The percentage was determined by the number of Ki67 or P53 positive cells among the total number of counted tumor cells. High expression of Ki67 was defined as ≥ 5%, P53 ≥ 10% as previously reported [33].

### Cell culture and Western blotting analysis

The breast cancer cell lines (MDA-MB-231 MCF-7), the cervical cancer cell line (Hela) and 293T cells were obtained from American Type Culture Collection. Cells were routinely maintained in high-glucose DMEM (Gibco, C11995) supplemented with 10% fetal bovine serum (Hyclone, SV30087.02), penicillin (100 units/mL; Sigma, P3032), and streptomycin (100 units/mL; Sigma, S9137) at 37°C in humidified 5% CO2 incubator. The expression of Bora in breast cancer and paired non-cancerous tissues was performed by western blot as previously reported [34].

Tissues and cells were lysed in RIPA buffer (50mM Tris pH 7.4, 150 mM NaCl, 1% NP-40, 0.5% sodium deoxycholate, 0.1% SDS, 1 mM phenylmethyl sulfonyl fluoride) containing protease inhibitor cocktail (Roche) and phosphatase inhibitor cocktail (Sigma-Aldrich). The protein concentration was detected by the Bradford method with BSA (Sigma-Aldrich) as the standard. Equal amounts of protein were subjected to SDS-PAGE and transferred to nitrocellulose membranes (Bio-Rad). The membranes were then blocked and incubated with antibodies against GAPDH (1:4000, Abmart, #M20006), Bora (1:1000, sigma, SAB3500025), Flag (1:5000, sigma, F1804), Plk1 (1:500, Santa Cruz Biotechnology, sc-17783), Phospho-Plk1 (Thr210) (1:1000, Cell Signaling Technology, 9062). After that, membranes were incubated with peroxidase-conjugated secondary antibodies (Millipore) and detected with enhanced chemiluminescence (Millipore) on X-ray films (Kodak).

### Plasmids, lentivirus production and transduction

pLVX-Flag (empty vector) was constructed by replacing DsRed gene in pLVX-DsRed-N1-Monomer (Clontech) with Flag sequence. Bora CDS was amplified from MDA-MB-231 cells and inserted into pLVX-Flag to construct pLVX-Bora-Flag. Lentivirus was produced in 293T cells using the second-generation packaging system plasmids psPAX2 (Addgene) and pMD2.G (Addgene) with Lipofectamine 2000. Infectious lentiviruses were collected at 48 h after transfection and filtered through 0.45-μm PVDF filters. MCF-7 cells were infected with concentrated virus in the presence of polybrene (8μg/ml, Sigma-Aldrich). The supernatant was replaced with complete culture medium after 24h, followed by selection with puromycin (2μg/ml), and the expression of Bora in infected cells was verified by western blot.

### RNA interference

siRNA oligonucleotides targeting Bora and non-targeting siRNA were purchased from GenePharma. Transfections with siRNA were performed with Lipofectamine 2000. The siRNAs against Bora were (1) 5′-CTATGAGACTTCAGATGTA-3′ and (2) 5′-TAACTAGTCCTTCGCCTAT-3′. The control siRNA (siNC) was 5′-TTCTCCGAACGTGTCACGGTT-3′.

### Synchronization

Cells were synchronized at prometaphase by a thymidine-nocodazole arrest (TN; a 18-hr thymidine arrest and a 5-hr release, followed by a 14-hr nocodazole arrest), then protein was extracted to detect the expression of Phospho-Plk1 (Thr210). Thymidine, 2.5 mM; Nocodazole, 100 ng/ml.

### Cell cycle and cell proliferation analysis

For cell cycle analysis, cells were collected and fixed in 70% ethanol overnight at 4°C. Fixed cells were incubated with 50 mg/mL propidium iodide and 100 mg/mL RNase at 37**°C** for 30 minutes and then analyzed by flow cytometry (Beckman Coulter).

For cell proliferation analysis, cells were seeded in 96-well flat-bottomed plates, with each well containing 1,000 cells in 100 μl of cell suspension. After a certain time in culture, cell viability was measured using Cell Counting Kit-8 (CCK-8) assays (Dojindo). Each experiment with five replicates was repeated three times.

### TUNEL assay

Cells were grown on glass coverslips, fixed with 4% paraformaldehyde solution and permeabilized with Triton X-100 (1%). For TUNEL assay, DNA strand breaks were detected with a TUNEL assay kit (KeyGEN BioTECH, KGA7051) according to the manufacturer's instructions. Nuclei were stained with DAPI. Merged images were counted under a confocal laser scanning microscope (OLYMPUS FV1000).

### Statistical analysis

Statistical analysis was performed using the SPSS software (SPSS standard version 16.0; SPSS Inc, Chicago, IL). The chi-square test was employed to evaluate the relationship between Bora and clinicopathologic characteristics. ROC curve analysis was performed to compare the sensitivity and specificity for the prediction of survival as described previously [35]. The relationship between Bora expression and survival was determined by Kaplan-Meier analysis. The log-rank tests were performed to evaluate the difference in survival probabilities between patient subsets. The Multivariate Cox proportional hazard models were utilized to estimate the Hazard Ratio (HR) and 95% confidence intervals (CI) for patient outcome. The comparison of means between two groups was conducted using Student's *t* test, whereas comparison for more than two groups was conducted using one-way ANOVA. All *P value*s quoted were two-tailed and *P* < 0.05 was considered statistically significant.

## SUPPLEMENTARY MATERIALS FIGURES


