# Supplementary material for: Cell cycle protein Bora serves as a novel poor prognostic factor in multiple adenocarcinomas
Source: Oncotarget. 2017 Mar 28;8(27):43838–52. doi: 10.18632/oncotarget.16631 (PMC5546444; doi:10.18632/oncotarget.16631)
Supplement: Supplementary file 1 [file oncotarget-08-43838-s001.pdf]

Cell cycle protein Bora serves as a novel poor prognostic factor in multiple adenocarcinomas

Supplementary Materials

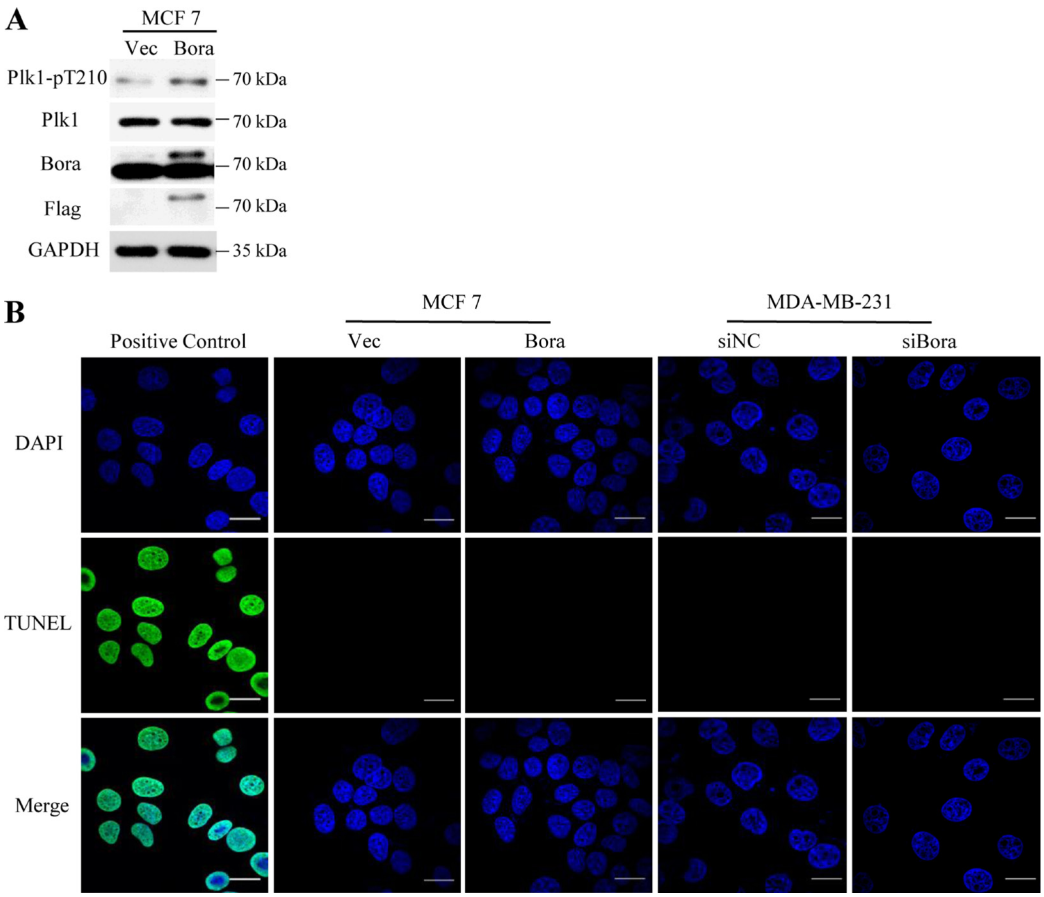

Supplementary Figure 1: Functional role of Bora in cell based assays. (**A**) Empty vector (Vec) or Flag-Bora (Bora) overexpressed MCF-7 cells were synchronized by a thymidine-nocodazole arrest and the indicated proteins were detected by Western blot. (**B**) Cells overexpressing Bora/Vec or transfected with siRNAs were assessed for DNA strand breaks by TUNEL assays (Green) according to the manufacturer's instructions. Nuclei were stained with DAPI (blue). DNase I treated sample was used as the positive control; Scale bars, 20 μm.
